# The effectiveness of interventions to improve laboratory requesting patterns among primary care physicians: a systematic review

**DOI:** 10.1186/s13012-015-0356-4

**Published:** 2015-12-05

**Authors:** Sharon L. Cadogan, John P. Browne, Colin P. Bradley, Mary R. Cahill

**Affiliations:** Department of Epidemiology and Public Health, University College Cork, Cork, Ireland; Department of General Practice, University College Cork, Cork, Ireland; Department of Haematology, Cork University Hospital, Cork, Ireland

**Keywords:** Interventions, Primary care, Behaviour change, Healthcare interventions, Laboratory testing

## Abstract

**Background:**

Laboratory testing is an integral part of day-to-day primary care practice, with approximately 30 % of patient encounters resulting in a request. However, research suggests that a large proportion of requests does not benefit patient care and is avoidable. The aim of this systematic review was to comprehensively search the literature for studies evaluating the effectiveness of interventions to improve primary care physician use of laboratory tests.

**Methods:**

A search of PubMed, Cochrane Library, Embase and Scopus (from inception to 09/02/14) was conducted. The following study designs were considered: systematic reviews, randomised controlled trials (RCTs), controlled clinical trials (CCTs), controlled before and after studies (CBAs) and interrupted time series analysis (ITSs). Studies were quality appraised using a modified version of the Effective Practice and Organisation of Care (EPOC) checklist. The population of interest was primary care physicians. Interventions were considered if they aimed to improve laboratory testing in primary care. The outcome of interest was a volume of laboratory tests.

**Results:**

In total, 6,166 titles and abstracts were reviewed, followed by 87 full texts. Of these, 11 papers were eligible for inclusion in the systematic review. This included four RCTs, six CBAs and one ITS study. The types of interventions examined included education, feedback, guidelines, education with feedback, feedback with guidelines and changing order forms. The quality of included studies varied with seven studies deemed to have a low risk of bias, three with unclear risk of bias and one with high risk of bias. All but one study found significant reductions in the volume of tests following the intervention, with effect sizes ranging from 1.2 to 60 %. Due to heterogeneity, meta-analysis was not performed.

**Conclusions:**

Interventions such as educational strategies, feedback and changing test order forms may improve the efficient use of laboratory tests in primary care; however, the level of evidence is quite low and the quality is poor. The reproducibility of findings from different laboratories is also difficult to ascertain from the literature. Some standardisation of both interventions and outcome measures is required to enable formal meta-analysis.

**Electronic supplementary material:**

The online version of this article (doi:10.1186/s13012-015-0356-4) contains supplementary material, which is available to authorized users.

## Background

Laboratory testing is an integral part of day-to-day practice in medicine and supports approximately 70 % of diagnoses and treatment decisions [1]. Further, among primary care physicians, an estimated 30 % of patient visits result in a laboratory request [2]. Healthcare budgets worldwide are facing increasing pressure to reduce costs and remove inefficiencies, while maintaining quality and safety. Laboratory testing is a major component of healthcare budgets in absolute terms, and demand for testing is increasing faster than medical activity [3]. In the National Health Service (NHS) in England, for example, an estimated £2.5 billion per annum is spent on laboratory services accounting for 3–4 % of the UK national health budget [4, 5]. Despite this relatively small proportion of healthcare budget expenditure, laboratory testing often underpins more costly downstream care such as outpatient visits and radiology requests.

The unnecessary use of laboratory services has been highlighted by a meta-analysis of 108 studies involving 1.6 million results from 46 of the 50 most commonly ordered lab tests in medicine [6]. This found that, on average, 30 % of all tests are likely to be unnecessary [6, 7]. With respect to primary care, US research has found that physicians order diagnostic laboratory tests for approximately 30 % of patient visits [8]. Authors reported that test-ordering factors including unnecessary test requests were responsible for 13 % of testing process errors in primary care [9].

The overuse of laboratory services can stem from the physician, the patient and the broader policy context. For example, some studies have found that many physicians report uncertainty over when to order tests and how to interpret test results [2]. Reasons given for this include lack of knowledge about indications, costs, insurance restrictions and inconsistent names for the same test [8]. Meanwhile, patients have high expectations that blood tests are performed and have little understanding of the limitations of testing [7, 10]. Other factors include a lack of knowledge regarding the financial effect of laboratory testing on the healthcare system [11] and the increasing volume of laboratory tests available to physicians [5]. Furthermore, system level factors associated with laboratory testing patterns have been identified and include limitations of laboratory and/or surgery information technology systems [5, 12]. As a result, it has been recommended that the theoretical and contextual factors responsible for changing primary care physician behaviour should also be considered when designing interventions [13–16].

A number of approaches for reducing unnecessary test ordering in primary care have implemented. These comprise of interventions aimed at tackling both the overutilization and underutilization of tests through strategies such as including cost displays on electronic order forms, facilitating educational workshops, and providing feedback to physicians on their test-ordering patterns [17–19]. However, the effectiveness of these strategies vary, and to date, no systematic reviews have focused solely on studies evaluating test-ordering behaviours of primary care physicians. Hence, the objective of this systematic review was to systematically and comprehensively search the literature for studies evaluating the effectiveness of interventions aimed at nudging primary care physicians’ ordering practice further in a direction which will maximally impact patient care. The Preferred Reporting Items for Systematic Reviews and Meta-Analyses (PRISMA) criteria guided reporting of the methods and findings (see Additional file 1).

## Methods

### Primary objective

The main objective of this systematic review was to synthesise the available published literature on interventions focused on improving the appropriateness of laboratory requesting patterns from primary care.

### Primary outcomes

The outcome of interest in this review was objectively measured provider performance (request rates or appropriateness of requests).

### Types of studies

Systematic reviews, randomised controlled trials (RCTs), non-randomised controlled trials (NRCTs), controlled before-after studies (CBAs) and interrupted time series analysis (ITSs) were considered for this review.

### Types of interventions

The review focused on interventions to change laboratory requesting patterns or improve laboratory requesting appropriateness.

### Data sources

The following databases were searched for potentially eligible studies: PubMed (1966 to Feb 9, 2014) the Cochrane Library (1993 to Feb 9, 2014), Embase (1974 to Feb 9, 2014) and Scopus (1960 to Feb 9, 2014). Updated searches of the electronic databases were performed in November 2014 to ensure additional relevant papers were not published since.

### Inclusion criteria

This review included interventions aimed at improving laboratory requesting patterns where objectively measured provider performance (requesting rates or appropriateness of requests) served as the dependent variable. Intervention studies were only considered if participants were primary care physicians, defined as any medically qualified physician providing primary healthcare and including general practitioners, family doctors, family physicians or family practitioners.

### Search strategy

PubMed was searched for potentially eligible studies by combining relevant medical subject headings (MeSH terms) with subheadings and text words (e.g. “utilisation”, “laboratory test(s)”). Only citations on human subjects were included. Search terms and search findings are provided in Additional file 2. For completeness, searches were repeated without subheadings and the results of these two searches were combined. The same methods were used for searching the Cochrane Library, Embase (Elsevier) and Scopus databases. Electronic searches were supplemented by cross-checking the reference lists of all identified studies. Duplicate citations were identified and removed using Endnote citation manager.

### Data collection and analysis

SLC carried out the electronic database searches. The search strategy for the review can be found in Additional file 2. Titles and abstracts of studies retrieved from the search strategy were reviewed independently by applying the appropriate inclusion/exclusion criteria. For each citation, two investigators (SLC and MRC) independently screened the titles and abstracts for potential relevance. The full text article was obtained for all potentially eligible studies. Any disagreements between SLC and MRC were resolved through discussion with a third reviewer (JPB).

Data were extracted from the included papers by a single reviewer (SLC). A second reviewer (JPB) checked data extraction sheets for errors. Information was extracted on study design, year of study, setting, participants, intervention characteristics and the reporting of results. A sample data extraction form can be found in Additional file 3.

It was not deemed appropriate to conduct a meta-analysis due to the heterogeneity of interventions and outcomes across the included studies. Instead, the existing analyses reported in the articles reviewed were extracted and reported in a narrative format.

### Quality assessment of included studies

Included studies were independently assessed for quality and risk of bias by SLC and JPB, with any disagreements resolved by discussion. This was performed using a modified version of the Cochrane Effective Practice and Organisation of Care (EPOC) Data Collection Checklist and Quality Criteria for studies with a control group (RCTs, CCTs and CBAs) and for ITSs studies [20]. The tool is specifically designed for interventions aiming to improve practice and provides a risk of bias assessment for each of the included study designs (RCT, CCT, CBA and ITS). This comprises of nine quality standards for RCTs, CCTs and CBAs: generation of allocation sequence, concealment of allocation, baseline outcome measurements, baseline characteristics, incomplete outcome data, blinding of outcome assessor and protection against contamination, selective outcome reporting and other risks of bias. For ITS study designs, the following three quality standards were also assessed: the independence of the intervention from other changes, the pre-specified shape of the intervention and if the intervention was unlikely to affect data collection [20].

## Results

### Search results

**Fig. 1:**
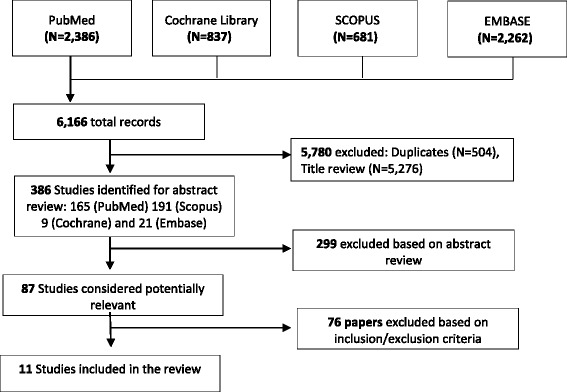
Flow diagram of the search strategy for review

In total, 6,166 records of papers were identified from the search of the literature (Fig. 1). Based on a title review, 5,276 records were excluded. A further 504 records were duplicates and also excluded. Of the 386 records remaining, 299 were excluded based on abstract review. Full texts were obtained for the remaining 87 records, of which 11 papers met the inclusion criteria and were included in the review (Table 1).

**Table 1:** Overview of intervention characteristics and results

Reference	Setting	Design	Participants	Type	Intervention	Comparator	Follow-up	Effect of intervention
Horn et al. [17]	USA	ITS	215 primary care physicians (5 group practices)	Changing order form	Cost displays within electronic health record at time of ordering (153 physicians)	Control group: no cost information (62 physicians)	12 months pre- and 6 months post-intervention	Difference-in-difference approach. 1–2.6 % reduction. 20 % The cost displays resulted in a reduction of 0.4–5.6 laboratory orders per 1000 visits per month (*p* < 0.001)
Kahan et al. [26]	Israel	CBA	Not disclosed	Changing order form	A new version of electronic order form	Older version of computerised order form	6 months pre- and 4 months post-intervention	31–41 % reduction relative to the pre-intervention month, with 36–58 % reduction the following month. −2–3 % changes for control tests
Shalev et al. [27]	Israel	CBA	865 primary care physicians	Changing order form	Changing volume of tests on order form (27 tests removed and 2 tests added—reducing the number of tests available using a check-box form from 51 to 26)	Standard form prior to intervention	12 months pre- and 24 months post-intervention	For deleted tests, there was a 27 % and 19.2 % reduction 1 and 2 years after intervention, respectively
Zaat et al. [46]	Netherlands	CBA	75 primary care physicians	Changing order form	Volume of tests on order form reduced (hand written request if test not displayed) (47 physicians)	Standard form (28 physicians)	Five month pre-intervention (control) and 12 months post-intervention	18 % reduction in number of tests requested monthly in experimental group after the intervention compared to the control doctors
Barrichi et al. [25]	Italy	CBA	44 primary care physicians	Education	Pathology-specific laboratory algorithms for 7 common clinical scenarios were tested. Education was provided (8 training sessions) to the physicians about the algorithms and their use (23 physicians)	Current practice	12 months pre- and 12 months post-intervention (data on test requests for randomly selected 30 days in each period)	5 % reduction in the volume of tests requested by the intervention district 1 year following the intervention (retrospective audit) compared with a 1 % increase in the control district
Larsson et al. [18]	Sweden	CBA	63 primary care physicians (19 practices)	Education	An education programme (2-day lecture series)	Current practice (2 practices)	5 months pre-intervention and 4 months post-intervention	7 ratios were recommended to decrease in volume, 5 did at *p* < 0.05. 7 were expected to increase in volume, 4 did at *p* < 0.05
Verstappen et al. [21]	Netherlands	RCT	174 primary care physicians (26 practices)	Education	A primary care physician-based strategy focused on clinical problems and associated tests (85 physicians in arm a and 89 physicians in arm b)	Each group acted as a control for the other	6 months pre- and 6 months post-intervention	12 % reduction in volume of total tests in intervention group versus no change in control arm. 16 % reduction of inappropriate tests for intervention group
van Wijk et al. [22]	Netherlands	RCT	60 primary care physicians (44 practices)	Guidelines	Guideline-based order form (29 GPs) versus restricted guideline-based electronic order form (31 GPs)	Each group acted as a control for the other	Study period: 1 July 1994–30 June 1995	Decision support based on guidelines is more effective in changing blood test ordering than decision support based on initially displaying a limited number of tests. Primary care physicians who used BloodLink-Guideline requested 20 % fewer tests on average than did practitioners who used BloodLink-Restricted (mean (±SD), 5.5 ± 0.9 tests versus 6.9 ± 1.6 tests (*p* = 0.003))
Baker et al. [23]	UK	RCT	96 primary care physicians (33 practices)	Guidelines and feedback	58 GPs (17 practices) guidelines followed by feedback about the numbers of thyroid function, rheumatoid factor test and urine cultures they ordered (quarterly for 1 year)	38 GPs (16 practices) received guidelines then feedback about lipid and plasma viscosity tests (each a control group for the other)	Baseline and 1 year post-intervention	No effect. No change in volume of tests per 1000 requested in either of the study groups for any of the tests
Thomas et al. [19]	UK	RCT	370 primary care physicians (85 practices)	Feedback and education	Quarterly feedback of requesting rates and reminder messages. Practices allocated to 1 of 4 groups: control (20 practices), enhanced feedback alone (22 practices), reminder messages alone (22 practices) or both enhanced feedback and reminder messages (21 practices)	Current practice	12 months pre- and post-intervention	11 % reduction in requests for practices receiving enhanced feedback or reminder messages (OR 0.89, 95 % CI 0.83–0.93) compared with control group
Tomlin et al. [24]	New Zealand	CBA	3160, 3140 and 3335 primary care physicians	Guidelines, feedback and education	3 marketing programmes (guidelines, individual feedback and professional development)	Locum and other physicians not targeted by the programmes	2 years pre- and post-intervention	60 % reduction in number of ESR tests by the intervention group following the intervention versus an 18 % reduction in comparison doctors after intervention

### Risk of bias in included studies

Table 2 provides details on the quality assessment of the studies. Ten of the 11 studies were deemed to have a high risk of bias, while one study [21] was deemed to be of low risk. Randomisation and allocation concealment was adequately performed for the four included RCTs [19, 21–23]. The most common reason for high risk of bias was the fact that participants (primary care physicians) in the intervention groups could not be blinded. This introduces the risk of information bias as physicians may have altered their requesting behaviours based on knowledge of being assessed. Only one of the studies adequately blinded participants [21]. Also, a key limitation of the RCT by Thomas and colleagues [19] was the lack of power to detect interactions and, hence, a much larger trial would be required to ensure that these interventions act independently. A key limitation of the included ITS study [17] was the physician-level design utilised with an absence of individual patient characteristics.

**Table 2:** Quality assessment of included studies

Author	Intervention	Design	Independent of other changes	Knowledge of allocated intervention	Unlikely to affect data collection	Shape of effect pre-specified	Attrition bias	Selective reporting	Other risk of bias	Overall risk
Horn et al. [17]	Changing order form	ITS	Low risk	High risk	Low risk	Low risk	Unclear risk	Low risk	Low risk	High risk
			The study had a control group	Participants knew which intervention they were receiving	Sources and data collection methods same before and after intervention	Specified	Missing data unclear	Appropriate outcomes reported	No other potential bias	
Author	Intervention	Design	Random sequence generation	Allocation concealment	Protection against contamination	Blinding	Attrition bias	Selective reporting	Similar at baseline	Overall risk
Shalev et al. [27]	Changing order form	CBA	High risk	High risk	N/A	High risk	Low risk	Low risk	N/A	High risk
			CBA study	CBA study	No control group	No blinding	No missing data	Appropriate outcomes reported	No control group	
Kahan et al. [26]	Changing order form	CBA	High risk	High risk	N/A	High risk	Low risk	Low risk	N/A	High risk
			CBA study	CBA study	No control group	No blinding	No missing data	Appropriate outcomes reported	No control group	
Zaat et al. [28]	Changing order form	CBA	High risk	High risk	Unclear risk	High risk	Low risk	Low risk	Unclear risk	High risk
			CBA study	CBA study	No information in text	Participants knew what they had been allocated to	No missing data	Appropriate outcomes reported	No information in text on baseline characteristics, baseline outcomes similar (Fig 1/2)	
Baricchi et al. [25]	Education	CBA	High risk	High risk	Unclear risk	High risk	Low risk	Low risk	Unclear risk	High risk
			CBA study	CBA study	No information in text	Participants knew what they had been allocated to	No missing data	Appropriate outcomes reported	No information in text	
Larson et al. [18]	Education	CBA	High risk	High risk	Unclear risk	High risk	Low risk	Low risk	Unclear risk	High risk
			CBA study	CBA study	No information in text	Participants knew what they had been allocated to	No missing data	Appropriate outcomes reported	No information in text	
Verstappen et al. [21]	Education	RCT	Low risk	Low risk	Low risk	Low risk	Low risk	Low risk	Low risk	Low risk
			Blocked randomization	Cluster trial, allocation after recruitment completed	Independent clinics	Controls were blinded, hence preventing the Hawthorne effect	No missing data	Appropriate outcomes reported	Outcomes measured at baseline and baseline characteristics reported and similar	
van Wijk et al. [22]	Guidelines	RCT	Low risk	Low risk	Low risk	High risk	Low risk	Low risk	Low risk	High risk
			“researcher not involved in the study…performed the randomisation using random-numbers table”	“each practice assigned by simple random allocation”	Separate practices	All participants knew what they had been allocated to. Test ordering in controls may have been affected	Missing data similar between groups and all participants accounted for	Appropriate outcomes reported	Similar baseline characteristics	
Baker et al. [23]	Guidelines and feedback	RCT	Low risk	Low risk	Low risk	High risk	Low risk	Low risk	High risk	High risk
			Random number table	Cluster trial, allocation after recruitment completed	Primary care physicians work separately	Participants knew what they had been allocated to. Test ordering in controls may have been affected	No sites lost to follow-up	Appropriate outcomes reported	Participants in group 2 had fewer patients and GPs than group 1	
Thomas et al.[19]	Feedback and education	Cluster RCT	Low risk	Low risk	Low risk	High risk	Low risk	Low risk	Low risk	High risk
			“cluster randomization…with a minimization procedure”	Cluster trial, allocation after recruitment completed.	Separate practices	Participants knew what they had been allocated to. Test ordering in controls may have been affected.	No missing data	Appropriate outcomes reported	Similar baseline characteristics	
Tomlin et al. [24]	Guidelines, feedback and Education	CBA	High risk	High risk	High risk	High risk	Low risk	Low risk	High risk	High risk
			CBA study	CBA study	“Changes…might be explained by ….contamination of the comparison group”	Participants knew what they had been allocated to. Test ordering in controls may have been affected	No missing data	Appropriate outcomes reported	Intervention group included GPs only while control group included locum GPs also	

### Characteristics of studies included in the review

Characteristics and the key findings of the included studies are presented in Table 1. Four of the studies were RCTs [19, 21–23], six were before-after studies [18, 24–28] and one was an ITS [17] with a parallel control group. The included studies were conducted in the Netherlands [21, 22, 28], USA [17], UK [19, 23], Italy [25], Israel [26, 27], Sweden [18] and New Zealand [24] with samples ranging in size from 44 to over 3,000 primary care physicians. The interventions covered by the review include education programmes [18, 21], laboratory profiles [25] clinical guidelines [22], guidelines and feedback combined [23], cost displays [17], the redesign of order forms [26–28] and the use of feedback and education strategies [19, 24].

### Clinical guidelines and policy recommendations

In their RCT study, van Wijk et al. [22] found that decision support based on guidelines integrated with patient electronic records was more effective for changing blood test-ordering behaviour than decision support based on limited testing offered in modified request forms. Primary care physicians who had access to the guideline-based system has ordered 20 % fewer tests per form than did primary care physicians who had access to the restricted system (mean ± SD 5.5 ± 0.9 versus 6.9 ± 1.6 tests, respectively; *p* = 0.003, Mann-Whitney test) [22]. Similar findings were obtained in the adjusted multivariate regression analysis [22]. Controlling for practice characteristics and historic test-ordering behaviour, 19 % more tests were requested by primary care physicians with access to the restricted order form (RR 1.19, 95 % CI 1.10–1.29) [22]. The study also reported a difference in requesting patterns between the two groups for specific tests. For example, in the restricted group, 61.2 % of order forms included an erythrocyte sedimentation rate test, compared with 44.1 % in the guideline group (*p* < 0.001) [22].

In a study using a CBA design and involving over 3,000 primary care physicians in New Zealand, Tomlin et al. [24] assessed the effect of three different marketing programmes promoting clinical guidelines. Each of these programmes involved written material advising of guideline recommendations. Individual laboratory-test use feedback data was distributed to each practice, and professional development opportunities were provided. The study found that clear information marketed to primary care physicians improved the quality of laboratory test ordering [24]. Some key findings included a 42 % reduction in erythrocyte sedimentation rate tests following the intervention (intervention physicians −60 %, comparison physicians −18 %, *p* < 0.01) [24].

### Feedback and reminders

Baker et al. [23] evaluated the use of feedback following guidelines in their RCT. Both groups received guidelines followed up with feedback about their use of selected tests. The first group of practices received feedback about their testing for thyroid function tests, rheumatoid factor and urine culture requests, while the second group received feedback about their serum lipids and viscosity requests. Hence, both groups were intervention groups but acted as control groups for the other group. The authors reported no change in laboratory requests quarterly feedback over a 1-year time period for any of the five tests studied [23].

A multifaceted clustered RCT by Verstappen et al. [21] aimed to optimise primary care physicians’ test-ordering behaviour by means of practice-based strategies targeting tests for specific clinical problems. Thirteen groups of primary care physicians underwent the strategy for three clinical problems (arm A; cardiovascular topics, upper and lower abdominal complaints), while 13 other groups underwent the strategy for three other clinical problems (arm B; chronic obstructive pulmonary disease and asthma, general complaints, degenerative joint complaints). The strategy consisted of personalised graphical feedback, including a comparison of each physician’s own data with those of colleagues; dissemination of national, evidence-based guidelines; and regular meetings on quality improvement in small groups [21]. Each of the arms of the trial acted as a control for each other. Physicians discussed personal feedback reports in small group meetings, related them to evidence-based clinical guidelines, and made plans for change. Authors reported a 12 % reduction in volume of total tests for arm A in intervention group versus no change in control arm (*p* < 0.001) [21]. However, for arm B of the trial, no statistically significant changes were identified (*p* = 0.22) [21].

In a third RCT by Thomas et al. [19], the use of feedback combined with educational reminder interventions was assessed. The feedback intervention involved the use of a booklet containing graphical presentations of individual practice level ordering for the targeted tests. Each practice was compared to regional statistics over a 3-year period. Educational reminders were developed in conjunction with the primary care physicians and were included with test results. The study found that primary care practices receiving either or both feedback and reminders had significantly reduced blood test utilisation (*p* < 0.001) [19]. The combined effect of the interventions resulted in a 22 % reduction in total number of targeted tests ordered (OR = 0.78, 95 % CI 0.71–0.85) versus 13 % for reminders alone (OR = 0.87, 95 % CI 0.81–0.94, *p* = <0.001) and 11 % for feedback alone (OR = 0.89, 95 % CI 0.83–0.94, *p* = 0.003) [19]. However, feedback led to greater reductions in the number of laboratory tests ordered compared with reminders although the model-based analyses suggested similar effects (adjusted change relative to baseline performance in audit and feedback arm = 12 %; OR for reminders = 0.89, 95 % CI 0.83 to 0.93, *p* = 0.003) [19].

### Education-based strategies

Baricchi et al. [25] evaluated the effect of seven pathology-specific laboratory profiles for more effective test requesting, using a CBA study design. Training sessions were facilitated to educate the primary care physicians in the intervention group about these profiles and to discuss their presumed usefulness. Authors reported a 5 % reduction in the volume of tests requested by the intervention group 1 year following the intervention compared with a 1 % increase in the control group (*p* = <0.001) [25].

Also using a CBA study, Larsson et al. [18] assessed the effects of an education programme which involved a 2-day lecture series at which each participant received a folder containing information relating to the guidelines for future reference. The authors reported significant changes (*p* < 0.05) for nine of 14 tests [18]. They recommended that ordering rates for seven ratios should decrease, of which five did (*p* < 0.05) and that seven ratios should increase in volume, of which four did (*p* < 0.05). None of the ratios significantly changed in the wrong direction [18].

### Cost displays of pricing information

Using an ITS design, Horn et al. [17] evaluated the effect of implementing cost displays for laboratory tests in primary care. The authors reported a reduction (1–2.6 %) in the volume of tests ordered for five out of 27 different laboratory tests when real-time display of cost information was provided electronically on patient record and results (estimated reduction of between 0.4/1000 visits per month and 5.6/1000 visits per month, *p* < 0.001) [17]. However, for higher cost tests, a reduction in test requests was observed in only one of six such tests [17].

### Changing order forms

Kahan et al. [26] evaluated a new version of a computerised order form for three target tests (vitamin B12, folic acid and ferritin) using a CBA study. Test requests for haemoglobin and iron were evaluated as controls. The authors reported a 31–41 % decrease in volume of requests for the three target tests at 1-month follow-up, with a further decrease to 36–53 % 2 months after the intervention [26]. In comparison, the effect on test requests for controls tests ranged from −2 to 3 % [26]. A second CBA study in Israel by Shalev et al. [27] evaluated changing the format of the existing check-box laboratory order form that is embedded in a computerised medical record. This involved removing 26 tests from the form and adding two tests. They found that for deleted tests, there was a 27 and 19.2 % reduction 1 and 2 years after intervention, respectively (*p* < 0.001) [27]. For unchanged tests, the percentage changes were +18.4 % in year one and −22.4 % in year two. Meanwhile, a 60.7 % (year one) and 90 % (year two) increase in volume was found where tests were added to the order form (*p* < 0.001) [27].

In a second CBA study, Zaat et al. [28] modified the request form so that it only had 15 tests listed and all other tests had to be hand written. The form also required more information about the reason for requesting the test. Primary care physicians in the intervention group received a copy of a booklet with descriptions of the essential characteristics of the 15 important tests on the new form. The authors reported an 18 % reduction in volume of laboratory test requested on a monthly basis for the intervention group [28]. In the comparison period, the difference between groups was significant (*p* < 0.0001) [28].

## Discussion

This review aimed to identify and evaluate interventions for improving the use of laboratory tests among primary care physicians. Intervention strategies included: education, feedback, guidelines, cost displays and changing the content of order forms. While included studies differed considerably in relation to the tests they assessed, the findings were consistently in the same direction, perhaps indicating some publication bias. All but one [23] of the included studies reported positive effects on laboratory testing patterns. However, a number of the studies included in this review have a high risk of bias and are lacking in certain areas of methodological quality.

Education-based interventions appear to have promising effects on improving primary care physician laboratory testing patterns in this review [18, 21, 25]. This included evidence from a high-quality RCT [21]. Similar educational strategies have also been effective in changing other primary care behaviours including improving prescribing patterns for antibiotics [29] and referral for radiological assessments [30]. In particular, diagnoses- or symptom-based education strategies involving a multidisciplinary approach proved effective [21, 25]. The sustainability of education-based strategies is often questioned in the literature. However, follow-up of long term effects of the education programme implemented by Larsson et al. [18] found it can be achieved with regular re-enforcement [31].

Further, the literature suggests provider education is inexpensive and feasible for widespread delivery [32]. Larsson et al. [18] also reported direct laboratory cost savings of their education programme. Similarly, Verstappen et al. [33] evaluated the costs and cost reductions of their feedback- and education-based strategy [21]. However, as with many intervention studies, a lack of rigorous economic evaluation methods and poor clinical data are key limitations of studies attempting to describe the economic value of their behaviour change strategies.

The feedback-based interventions in this review were multifaceted, and their effects were dependent on the particular combination of strategies used [19, 23, 24]. Feedback strategies have also shown positive results for other test-ordering activities by primary care physicians such as electrocardiogram use [34, 35]. In particular, enhanced feedback combined with brief educational reminder messages had a positive effect on requesting patterns [19]. The broader literature also suggests that feedback interventions have improved success when combined with other education-based strategies, including outreach visits or educational reminders [36, 37]. In primary care, providing feedback may change attitudes towards current practice and subsequent clinical outcomes, by changing self-assessment or by directing attention to a particular set of guidelines [38].

However, Baker et al. found that feedback was ineffective for changing primary care physician requesting behaviour when provided following guidelines [23]. The literature suggests that this may be explained by baseline performance, how often feedback is provided and how the feedback is provided [38, 39]. Moreover, individualised feedback in other areas of clinical performance has been shown to be more effective than general feedback, in particular when it is regular and repeated [40].

Implementation strategies for the delivery of education-based strategies may also be important, in particular the dissemination of guidelines. Decision support based on limited testing offered in modified request forms was less effective compared to decision support based on guidelines integrated with patient electronic records for changing blood test-ordering behaviour [22]. However, the use of guidelines is often criticised with respect to sustainability of change in the long term [11].

Real-time display of cost information provided electronically on patient record, and results showed a significant but small change in laboratory testing patterns [17]. However, this change was dependent on specifics of the test with insignificant changes for five out of six of the high-cost tests [17]. Little research exists on the effectiveness of cost displays for altering behaviour in the primary care; however, conflicting evidence exists among studies that have included physicians in hospital settings [41, 42]. In addition, diverse background health systems need to be considered when implementing a cost-display-based strategy.

### Implications for the implementation of interventions

Some multifaceted intervention strategies within the scope of this review have shown positive results although conflicting evidence exists in the wider literature on changing healthcare professional behaviours [43, 44]. Grimshaw and colleagues highlight that few studies explain the rationale for choice of a particular combination of interventions [44]. The authors conclude that if interventions are tailored to address local barriers to change, then multifaceted approaches may be more effective than individual interventions [44]. There is a literature to support the belief that knowledge of pre-requesting variables contributes to the success of interventions in health professionals [45]. For example, some authors identified test decision points including primary care physicians’ preference for risk [46] and perceived needs of the patient for reassurance [47]. Studies have also highlighted the importance of factors associated with wider health system performance [46].

Interestingly, in this review, none of the interventions appear to be designed based on the attitudes and behaviours responsible for laboratory testing patterns. The use of theory to understand such barriers has been recommended for the design of behaviour change interventions [13, 14]. In particular, Michie and colleagues have highlighted the importance of understanding the nature of the behaviour to be changed and the context [16]. The authors argue that designing interventions based on practitioner or researcher intuition rather than theory prevents an understanding of the behaviour change processes responsible for effective interventions [16]. To address this issue, they have developed a behaviour change theory, the “capability, opportunity, motivation-behaviour (COM-B) system”, which can be used as a taxonomy to map any identified barriers to the origin of the problem [15]. Hence, implementation strategies should also consider the theoretical and contextual factors responsible for changing primary care physician behaviour when designing interventions.

Similarly, the wider impacts of these interventions on the clinical outcome and the management of the patient need to be considered. There is a paucity of data on downstream effects of laboratory ordering. Few studies have attempted to link and quantify laboratory ordering with subsequent ordering of radiology or outpatient requests [48], and none have linked a reduction of laboratory orders with reduced follow on requests. Further research on the proportion of laboratory requests, where the result (either normal or abnormal) leads to a quantifiable diagnosis, health gain or evidenced-based health intervention, is required. Ultimately, the laboratory mission is to serve the patient and most studies to date have focused on the requester. Thus, while laboratory-based interventions to curtail inappropriate requests are valuable, they rarely have a patient focus. Aiming to reduce the volume of test requests may not be a satisfactory outcome of interest. The key drivers of demand management and improving the appropriateness of test requesting should include economic savings in the health service but also improved clinical outcomes for patients [5, 12]. In order to do so, a collaborative approach involving laboratories and clinicians may be most beneficial. For example, provision of interpretative comments on test reports is not only welcomed by users but has been suggested to influence requesting behaviour and, indeed, patient outcomes.

### Limitations of this review

Firstly, the heterogeneity of the studies precludes a quantitative pooling of the results to produce any statistical inference; our study is thus essentially descriptive. Secondly, follow-up periods of the included studies varied, ranging from 3 months to 2 years. As a result, the findings may vary. Also, this review followed EPOC adapted guidelines when including study designs and included a mixture of RCT, CBA and ITS study designs. The latter two study designs are weaker and more susceptible to bias due to their observational nature. Finally, not all included studies controlled for the same confounders, in particular patient-level characteristics.

Another limitation of our review is that it is possible that additional studies with non-significant or negative effects were not published. This leads to publication bias which may have an impact on our findings. In particular, clinical laboratories may be carrying out routine audits of new strategies they have implemented but may be not publishing less favourable negative results. Also, in many of the included studies, authors reported a suspicion of inappropriate testing as part of their motivation [17, 19, 21–23, 25, 27, 28].

However, our study also has several strengths. Our literature search is exhaustive and provides a clear overview of the subject matter. The studies included are from practices within covering multiple geographic locations; thereby, the inferences of the review are generalisable to a large population.

## Conclusions

Our review suggests that many different interventions may change primary care physician requesting patterns, in particular multifaceted interventions. However, due to the small number of studies and questionable validity and generalisability of findings of these studies, this review should encourage further better quality research in this area. While some of the included study designs are weak, the results are generally consistent in pointing to the need to intervene to improve test-ordering behaviour. As a result, it is important that policy makers consider the benefits of providing the resources to further explore and implement some of these interventions pending the conduct of better quality studies. The possibility of publication bias should be weighed when assessing interventions. However, due to the downstream expenditure resulting from laboratory testing, the cost and time associated with continuous quality improvement initiatives in laboratory medicine will be beneficial.

There is a paucity of theory-based interventions in relation to test-ordering behaviours of physicians. Further research should concentrate on improving our understanding of when interventions such as education or guidelines are likely to be effective and how to improve them. In particular, current interventions have been limited to tackling only one or a very few elements of the behaviour change wheel. As a result, the determinants of success and failure remain unclear and interventions may not be applicable to specific tests. Given the difficulties inherent in translating research into practice, it is reasonable to question whether the interventions we describe are generalisable or adaptable to other healthcare settings and conditions. Also, future studies that examine the effect of combined approaches, conducted by a multidisciplinary team are likely to be of interest. Hence, further research is needed to systematically examine the contextual and organisational factors likely to influence the behaviour change and implementation. In addition, research focused on the impact on patient care, further testing and diagnosis as a result of change in laboratory ordering would assist with appropriate policy for future laboratory services.

## Additional files

Additional file 1:
**PRISMA checklist**. Completed PRISMA checklist indicating page number in manuscript of relevant content. Additional file 2:
**Search terms and search records for PubMed, Cochrane, Embase and Scopus databases**. Additional file 3:
**Sample data extraction form**.

## Abbreviations

CBA: controlled before and after study
CCT: controlled clinical trial
EPOC: Effective Practice and Organisation of Care
ITS: interrupted time series
NRCT: non-randomised controlled trials
RCT: randomised controlled trial
