# Supplementary material for: The effectiveness of interventions to improve laboratory requesting patterns among primary care physicians: a systematic review
Source: Implement Sci. 2015 Dec 5;10:167. doi: 10.1186/s13012-015-0356-4 (PMC4670500; doi:10.1186/s13012-015-0356-4)
Supplement: Additional file 1: — PRISMA checklist. Completed PRISMA checklist indicating page number in manuscript of relevant content. [file 13012_2015_356_MOESM1_ESM.docx]

| S1: **Search terms and search records for PubMed, Cochrane, Embase and Scopus databases** | | | | | | |
| --- | --- | --- | --- | --- | --- | --- |
| **Search terms*** | **Database^+^** | | | |  | |
|  | *Scopus (Feb 09^th^ 2014) LIMIT: humans* | *EMBASE (Feb 09^th^ 2014) LIMIT: humans & English language* | *Cochrane (1981-Feb 09th 2014 ) LIMIT: humans* | *PubMed (1981-Feb 09th 2014) LIMIT: humans* | |  |
|  | **records returned** | **records returned** | **records returned** | **records returned** | |  |
| 1. Laboratory test | 355,218 | 193,805 | 7,536 | 92,822 | |  |
| 1. Laboratory tests | 355,218 | 59,254 | 7,536 | 77,746 | |  |
| 1. Laboratory testing | 109,218 | 52,719 | 7,531 | 37,044 | |  |
| 1. Clinical laboratory tests | 140,878 | 42,669 | 4,466 | 36,286 | |  |
| 1. Laboratory requests | 3,280 | 1,052 | 117 | 753 | |  |
| 1. Laboratory orders | 79,088 | 1,467 | 1,135 | 1,219 | |  |
| 1. Clinical laboratory requests | 1,476 | 785 | 65 | 429 | |  |
| 1. Test orders | 332,695 | 1,253 | 9,244 | 1,829 | |  |
| 1. Laboratory use | 1,105,378 | 103,694 | 7,952 | 566,999 | |  |
| 1. Laboratory test utilization | 4,928 | 1,842 | 76 | 1,731 | |  |
| 1. Laboratory test requests | 1,561 | 484 | 75 | 297 | |  |
| 1. Lab requests | 367 | 232 | 5 | 95 | |  |
| 1. #1#2#3#4#5#6#7#8#9#10#11#12 | 904 | 310,976 | 20,635 | 568,963 | |  |
| 1. Physician* | 533,906 | 429,285 | 14895 | 379,721 | |  |
| 1. Family physician | 74,269 | 70,155 | 1942 | 55,359 | |  |
| 1. Family physicians | 74,269 | 59,685 | 1942 | 36,904 | |  |
| 1. Family doctor | 30,996 | 45,788 | 565 | 41,742 | |  |
| 1. Family doctors | 30,996 | 7694 | 565 | 45,361 | |  |
| 1. Primary care physician | 53,707 | 46,722 | 888 | 14,235 | |  |
| 1. Primary care practice | 80,473 | 72,714 | 559 | 46,756 | |  |
| 1. Primary care physicians | 53,707 | 50413 | 888 | 24,292 | |  |
| 1. General practitioners | 97,606 | 38,029 | 2,877 | 28,285 | |  |
| 1. General practitioner | 97,606 | 90,422 | 2,877 | 30,719 | |  |
| 1. General practice | 206,566 | 149,377 | 3607 | 114,625 | |  |
| 1. Primary care doctors | 14,931 | 6,299 | 28 | 30,123 | |  |
| 1. Primary health care | 178,733 | 177,459 | 3,447 | 137,689 | |  |
| 1. Primary care provider | 18,856 | 8,065 | 323 | 5,963 | |  |
| 1. Primary care providers | 18,856 | 14150 | 323 | 11,314 | |  |
| 1. #14#15#16#17#18#19#20#21#22#23#24#25#26#27#28 | 18,768 | 700,614 | 29,622 | 570,365 | |  |
| 1. intervention | 733,111 | 386,906 | 80,333 | 282,989 | |  |
| 1. Strategy | 1,384,949 | 188,906 | 21706 | 159,097 | |  |
| 1. Inappropriate | 69,485 | 37,868 | 11339 | 32,513 | |  |
| 1. Ordering performance | 6,465 | 695 | 2681 | 491 | |  |
| 1. Efficiency | 1,384,080 | 103,505 | 6633 | 283,575 | |  |
| 1. Volume, tests | 174,045 | 18,932 | 9794 | 35,253 | |  |
| 1. #30#31#32#33#34#35 | 9,759 | 18,952 | 112,391 | 594,617 | |  |
| 1. Trial | 157,8224 | 1,082,279 | 305342 | 915,699 | |  |
| 1. Article | 1,889,975 | 8,184,301 | 79285 | 11,905,601 | |  |
| 1. Audit | 65,946 | 38,002 | 1098 | 27,870 | |  |
| 1. Systematic review | 133,162 | 116,281 | 19396 | 1,588,086 | |  |
| 1. Before and after study | 3,097,342 | 300,765 | 54656 | 990,275 | |  |
| 1. #37#38#39#40#41 | 481,205 | 8,668,501 | 365251 | 11,976,838 | |  |
| 1. #13#29#36#42 ^ | **681** | **2,262** | **837** | **2,386** | |  |

**^+^** Updated searches performed on all databases November 2014

* Row 13: Search included laboratory test OR laboratory tests OR laboratory testing OR clinical laboratory tests OR laboratory requests OR laboratory orders OR clinical laboratory requests OR test orders OR laboratory use OR laboratory test utilization OR laboratory test requests or lab requests (combination of search terms for laboratory testing from rows 1-12)

^ Full search: all search terms for laboratory test (row 13) AND all search terms for primary care physician (row 29) AND all search terms for intervention characteristics (row 36) AND all search terms for study designs (row 42)
