# Supplementary material for: The effectiveness of interventions to improve laboratory requesting patterns among primary care physicians: a systematic review
Source: Implement Sci. 2015 Dec 5;10:167. doi: 10.1186/s13012-015-0356-4 (PMC4670500; doi:10.1186/s13012-015-0356-4)
Supplement: Additional file 2: — Search terms and search records for PubMed, Cochrane, Embase and Scopus databases. [file 13012_2015_356_MOESM2_ESM.docx]

**Supplementary file 2: Example data extraction form: Thomas et al 2006**

| **Setting**: Scotland, United Kingdom  **Number of General Practitioners:** 85 primary care practices (370 GPs)  **Number receiving intervention:**   - Feedback only: 22 practices - Reminders only: 22 practices - Both feedback and reminders: 21 practices - Control group: 20 practices | |
| --- | --- |
| **Study design, duration and follow-up**:  Cluster RCT, 2x2 factorial design.  Duration: 12 months (commenced in Feb 2002)  Data: 12 month pre intervention and 12 month intervention period | |
| **Intervention:**  Reminders group: Brief educational messages were added as reminders to the test result reports for nine laboratory tests sent to the requesting practice. The messages were activated by the laboratory system using cues and were presented at the same time as the test result.  Feedback group: Feedback was quarterly, and consisted of a six-sided colour booklet presenting graphs of practice level data for each of the nine targeted tests and for each laboratory discipline as a whole. Every graph showed rates of test requesting over the previous 3 years (number per 10 000 patients per 6 months) for the practice compared with the regional rates. The booklets were posted to each family practitioner within each intervention group practice on four occasions (updated every 3 months from the start of the intervention period).  Feedback and reminders group: The feedback was enhanced with the educational messages which were included alongside the graphs for each of the targeted tests. | **Control:**  Usual practice. |
| **Outcome measures:**   - Volume of laboratory tests ordered per practice were obtained for the 12 months before (pre-intervention) and during the 12 months of intervention period. | |
|  | |
| **Results:**  **Pre-intervention Post- intervention**   - Control group: 1,071 (783-1,804) 1,226 (726-2,057) - Feedback only: 1,233 (601-1,954) 1,079 (575-1,818) - Reminders only: 1,329 (688-1,726) 1,317 (719-1,590) - Both: 1,166 (492-1,749) 1,041 (362-1,515)   NOTE: Data are median (IQR) test requests per 10,000 patients per practice   - Practices that received either feedback or reminders were less likely than the control group to request the targeted tests in total (enhanced feedback OR: 0.87, 95%CI 0.81-0.94; p= 0.0004; reminder messages OR: 0.89, 95%CI: 0.83-0.93; p=0.003). - Practices that received the combined (feedback and reminders group) were also significantly less likely than the control practices to request the target tests (OR: 0.78, 95%CI: 0.71-0.85). - The effect varied across the target tests, although the general pattern was that of a reduction in test volumes. - The enhanced feedback strategy reduced test ordering for all nine tests, which reached statistical significance (P<0.05) for four tests.(antibody screen, FSH, TSH and vitamin B12). - The brief educational reminders messages showed a reduction in test requests for eight of the nine target tests, of which three reached statistical significance (carcino-embryonic antigen, TSH and vitamin B12) | |
| **Losses to follow up:** no losses to follow up | |
